# Mycophenolate Mofetil in a Lupus Patient with Pulmonary Hypertension

**DOI:** 10.7759/cureus.2121

**Published:** 2018-01-29

**Authors:** Kirthi Machireddy, Zin Myint, Eric Dein, Stephen C Mathai, Philip Seo, Uzma Haque, Rebecca Manno, Homa Timlin

**Affiliations:** 1 Other, University of the Sciences In Philadelphia; 2 Hematology & Oncology Division/university of Kentucky, University of Kentucky School of Medicine; 3 Internal Medicine/hopkins Bayview, Johns Hopkins Bayview Medical Center; 4 Division of Pulmonary and Critical Care Medicine, The Johns Hopkins University School of Medicine; 5 Division of Rheumatology, The Johns Hopkins University School of Medicine; 6 Medicine, The Johns Hopkins University School of Medicine

**Keywords:** mycophenolate mofetil, pulmonary hypertension, systemic lupus erythematosus

## Abstract

Pulmonary hypertension (PH) is a life-threatening complication of several, different connective tissue diseases, including systemic lupus erythematous (SLE), systemic sclerosis, and rheumatoid arthritis. PH can present early in SLE. The severity does not correlate with other organ disease activity or with disease duration. It is still debatable whether immunosuppressive therapy is useful for PH related to SLE or autoimmune connective tissue disease, as there are no large clinical trials. However, several case reports have shown improvement with cyclophosphamide and prednisone with or without vasodilator therapy. We present a case of SLE-related PH in which a dramatic improvement in mean pulmonary artery pressure and exercise capacity was noted after the institution of treatment with mycophenolate mofetil, resulting in a decrease in corticosteroid dose. Our observations support the potential value of mycophenolate mofetil therapy for PH in SLE.

## Introduction

Pulmonary hypertension (PH) is a severe, potentially life-threatening complication of autoimmune connective tissue diseases, particularly in systemic sclerosis (SSc) and systemic and lupus erythematous (SLE) [1]. Among lupus patients, PH can be caused by pulmonary arterial hypertension (similar to idiopathic pulmonary arterial hypertension (PAH)), thromboembolic disease, advanced interstitial lung disease with hypoxemia, and left ventricular dysfunction. In a recent, large registry from China, SLE was the most common connective tissue disease (CTD) associated with PH, and the reported prevalence of PH in SLE ranges from 0.5% to 14% [1]. The pathogenesis of connective tissue disease-associated PH is often a result of concomitant molecular and tissue-level factors [2], and immune complex deposition has been identified on transbronchial biopsy [3], which suggests an underlying inflammatory component.

While SLE-associated PH predominantly affects young women within the first five years of SLE diagnosis, it can occur at any time during the disease course and the prevalence and severity of PH in SLE does not correlate with disease activity in other organs or with disease duration [3]. The one-year survival of SLE patients with pulmonary hypertension has been reported to be 91%-92% whereas systemic sclerosis was 80%.

The mainstays of therapy for PH have included supplemental oxygen, anticoagulants, calcium channel blockers, prostacyclin (both infused and inhaled), selective and nonselective endothelial antagonists, and phosphodiesterase inhibitors. There is emerging evidence that intensive immunosuppressive therapy can be effective in patients with autoimmune connective tissue disease as compared to idiopathic PH [4]. We herein report a patient with lupus who developed PH early in her disease course and experienced significant improvement in her pulmonary hypertension with an immunosuppressive regimen that included mycophenolate mofetil and corticosteroids.

## Case presentation

A 27-year-old African-American woman presented with a one-week history of dull left-sided chest pain that radiated to her back and was relieved by leaning forward. She denied dyspnea or fever. Her physical examination was unremarkable; there was no pericardial rub. Serum troponin T was 0.079 ng/ml (normal < 0.045 ng/ml). Tests for urine cocaine metabolites were negative. By transthoracic echocardiography (TTE), she had a normal left ventricular function with an ejection fraction of 65%, a normal right ventricular size and function, and the absence of valvular abnormalities or pericardial effusion. She was treated with indomethacin for presumed viral pericarditis.

Three weeks later, she presented with a recurrence of her chest pain, now associated with presyncope. Transthoracic echocardiography findings were unchanged. She was prescribed colchicine for recurrent viral pericarditis.

Six weeks later, her symptoms had progressed; she was admitted to the hospital with dyspnea on exertion, recurrence of chest pain, and a new malar rash. She had positive tests for anti-nuclear antibodies (titer 1:2560, speckled pattern), anti-double-stranded DNA 234 IU and anti-ribonuclear antibodies, IgM anti-cardiolipin of 34, IgG anti-cardiolipin of 30, IgM and IgG anti-beta2 glycoprotein < 10, with negative lupus anticoagulant, anti-Scl70, and RNA polymerase III antibody. WBC was 2800/µl and platelet count was 100,000/ µl. Oral prednisone in tapering doses and hydroxychloroquine were prescribed for SLE-related pericarditis. Transthoracic echocardiography now documented severe PH with an estimated right ventricular systolic pressure (RVSP) of 92 mmHg and severe right ventricular dysfunction.

Two weeks after hospital discharge, she was readmitted with a headache, blurred vision, myalgia, generalized muscle weakness, difficulty walking, and hypertensive urgency (BP: 195/145 mmHg). Creatine kinase (CK) was 1293 units/L and CK-MB was 85.3 ng/ml (normal CK 30-170 units/L and normal female CK-MB < 3.8 ng/ml). There was no renal artery stenosis or evidence of abdominal vasculitis on MR angiography. Intracranial arteries were unremarkable without evidence of vasculitis on head computed CT angiography. Transthoracic echocardiography documented severe right ventricular dysfunction with an estimated right ventricular systolic pressure (RVSP) of 97 mmHg. On right heart catheterization (RHC), the right atrial pressure was 60 mmHg, pulmonary capillary wedge pressure was 11 mmHg, cardiac output was 4.9 L/min, cardiac index was 2.5 L/min/m^2^, and pulmonary vascular resistance was 10 Wood units. These data were consistent with severe precapillary PH with mildly impaired cardiac function. There was a low probability of pulmonary emboli by ventilation/perfusion scanning and no intraluminal pulmonary artery defects, lung parenchymal defects, or features of interstitial lung disease on computed tomography (CT) angiography. Cardiac magnetic resonance imaging (MRI) showed no evidence of myocarditis. Pulmonary function tests (PFTs) showed a moderate obstructive ventilatory defect with a moderate diffusion abnormality pattern; forced expiratory volume (FEV) was 2.62 L (86.6% predicted); FEV1 was 1.76 L (68% predicted); FEV1/forced vital capacity (FVC) was 67%; total lung capacity (TLC) was 3.5 L (83.9% predicted); residual volume right ventricle (RV) was 1.05 (87.4% predicted); and diffusing capacity of the lung (DLCO) was 47% predicted.

She was diagnosed with SLE-associated PH and polymyositis. During her hospital stay, she was treated with pulse dose methylprednisolone 1000 mg daily for five days. She was discharged on intravenous treprostinil for her PH and was on a prednisone dose of 60 mg daily at this time. During her outpatient follow-up, she had a malar rash and proximal muscle weakness without sclerodactyly or synovitis. She was initiated on mycophenolate mofetil 500 mg twice daily in addition to prednisone (which had been tapered to 10 mg daily over three months) given her proximal muscle weakness in the setting of PH. The patient did not proceed with electromyography (EMG) and thigh muscle MRI. Her labs revealed a negative myositis panel and negative antiphospholipid antibodies on repeat testing. Within one month, the mycophenolate mofetil was titrated up to 1500 mg twice daily and the prednisone dose was decreased to 7.5 mg daily. She showed an improvement in her leg weakness, with a decrease of her CK level from 1293 units/L to 385 units/L and improvement in her exercise capacity. A muscle biopsy was not performed given the improvement in her symptoms. On day 49, prednisone was decreased to 5 mg daily.

Repeat transthoracic echocardiography four months later showed a normal right ventricular size and function with an estimated RVSP of 29 mmHg. Repeat right heart catheterization on low-dose treprostinil (19 ng/kg/min) showed a reduction in the mean pulmonary artery pressure to 32 mmHg, an increase in cardiac output to 5.3 L/min, and a reduction in pulmonary vascular resistance to 4.2 Wood units. Since she was receiving only low-dose treprostinil for her PH, her significant symptomatic and hemodynamic improvement was felt to be related to augmented immunosuppression; subsequently, oral therapy with sildenafil was initiated and treprostinil was tapered until discontinuation.

## Discussion

We report a young African-American woman with lupus-related PH who responded to treatment with mycophenolate mofetil and corticosteroids.

The pathogenesis of PH is multifactorial, including vascular remodeling, perivascular inflammation, and endothelial dysfunction. Furthermore, pulmonary venous hypertension from left ventricular dysfunction, hypoxic vasoconstriction from chronic hypoxemic lung disease, thromboses related to antiphospholipid antibodies, and veno-occlusive processes related to the hypercoagulable state in SLE may contribute to the development of PH. A role for inflammation in SLE-related PH is suggested by the finding of macrophage and lymphocyte infiltrates in the vascular remodeling in most PH types [2].

In addition to this, several circulating autoantibodies are found in the serum of patients with SLE-related PH, suggesting a role for immune-mediated damage. Furthermore, Lian et al. found serositis, Raynaud’s phenomenon, high disease activity, anticardiolipin antibodies, and anti-U1RNP were significantly associated with SLE-related PH [5].

The mainstay of the treatment of PH includes oxygen, diuretics, anticoagulation, and pulmonary vasodilator therapy with endothelin receptor antagonists, phosphodiesterase-5-inhibitors, and/or prostacyclin analogs. In a review of the literature, Sanchez et al. identified 11 patients with connective tissue disease-related PH who were treated with immunosuppressive agents, including corticosteroids alone in five; improvement was noted in all six patients treated with corticosteroids and a second immunosuppressive agent but in only one of the five patients who received corticosteroids alone [4]. In a controlled trial of intermittent pulse cyclophosphamide versus oral enalapril in the treatment of PH in 34 patients with SLE, there was a greater decrease in the systolic pulmonary artery pressure among the cyclophosphamide-treated patients (P = 0.003), but enalapril was not effective (P = 0.14) [6]. In another single-center study, five of 12 SLE patients with PH responded to treatment with monthly intravenous cyclophosphamide alone or with glucocorticoids; none of the six systemic sclerosis patients responded [7].

In a retrospective analysis of 23 patients with PH associated with SLE or autoimmune connective tissue disease, 16 were treated with immunosuppressive therapy alone (intravenous cyclophosphamide monthly for six months and oral prednisone for four weeks), while seven were treated with both immunosuppressive and pulmonary vasodilator therapy [8]. There was a significant improvement in the mean pulmonary artery pressure in eight out of the 16 patients treated with immunosuppressive therapy alone. Among these eight responders, five maintained a stable clinical and hemodynamic status during a median follow-up period of 47 ± 30 months after the last dose of cyclophosphamide, while three relapsed and needed another course of cyclophosphamide therapy, resulting in severe cyclophosphamide toxicity. The role of monotherapy with steroids in lupus-related PH is limited [3]. Moreover, Perez and Kramer found a poor response to a combination of vasodilator and corticosteroid therapy [9].

Mycophenolate mofetil is an immunosuppressive agent originally used in solid organ transplantation and has been shown to inhibit vascular endothelial and smooth muscle cell proliferation. This can alleviate pulmonary arteriolar wall thickening and inhibit abnormal vascular remodeling in rodent models of PH [10].

## Conclusions

In conclusion, we observed a major improvement in SLE-related PH in this young patient with an early disease who was treated with mycophenolate mofetil.

Our observations support the potential value of mycophenolate mofetil therapy for pulmonary hypertension in SLE.
